# Integrated proteomic and metabolomic analysis to study the effects of spaceflight on *Candida albicans*

**DOI:** 10.1186/s12864-020-6476-5

**Published:** 2020-01-17

**Authors:** Jiaping Wang, Yu Liu, Guangxian Zhao, Jianyi Gao, Junlian Liu, Xiaorui Wu, Chong Xu, Yongzhi Li

**Affiliations:** China Astronaut Research and Training Center, Beijing, 100094 China

**Keywords:** Spaceflight, Metabolomics, Proteomics, *Candida albicans*

## Abstract

**Background:**

*Candida albicans* is an opportunistic pathogenic yeast, which could become pathogenic in various stressful environmental factors including the spaceflight environment. In this study, we aim to explore the phenotypic changes and possible mechanisms of *C. albicans* after exposure to spaceflight conditions.

**Results:**

The effect of *C. albicans* after carried on the “SJ-10” satellite for 12 days was evaluated by proliferation, morphology, environmental resistance and virulence experiment. The result showed that the proliferation rate, biofilm formation, antioxidant capacity, cytotoxicity and filamentous morphology of *C. albicans* were increased in the spaceflight group compared to the control group. Proteomics and metabolomics technologies were used to analyze the profiles of proteins and metabolites in *C. albicans* under spaceflight conditions. Proteomic analysis identified 548 up-regulated proteins involved in the ribosome, DNA replication, base excision repair and sulfur metabolism in the spaceflight group. Moreover, 332 down-regulated proteins related to metabolic processes were observed. The metabolomic analysis found five differentially expressed metabolites. The combined analysis of proteomic and metabolomic revealed the accumulation of cysteine and methionine in *C. albicans* after spaceflight.

**Conclusions:**

Mechanisms that could explain the results in the phenotypic experiment of *C. albicans* were found through proteomic and metabolomic analysis. And our data provide an important basis for the assessment of the risk that *C. albicans* could cause under spaceflight environment.

## Background

In recent years, with the development of space technology, microbial space safety has become a research hotspot. It has been known that microorganisms such as bacteria and fungi have widely existed in the International Space Station [1]. *C. albicans* is a common conditional pathogen that usually parasitizes on human skin, mouth, urinary tract, and reproductive system [2]. While the pathogenicity of *C. albicans* may change with the changes in the external environment [3]. It has been reported that microorganisms including *C. albicans* proliferate more rapidly in the International Space Station [4, 5], thereby multiplying the risk of onboard cross-contamination, colonization, and infection. Worse, the space environment can potentially alter microbial physiology and virulence [6]. Moreover, the immunological investigations of the astronauts recorded several dysregulations, including lymphocyte proliferation, cytokine production [7] and redistribution of leukocyte subsets [8]. Together, the presence of *C. albicans* poses a potential threat to astronauts’ health. However, little is known about the molecular mechanism changes of *C. albicans* under the space environment.

A comprehensive understanding of the molecular communication will therefore provide new insights into this molecular mechanism. With the development of omics technology, high-throughput molecular identification and quantitation became available. Proteomics [9] could explore the expression of all the proteins of *C. albicans* under different environmental conditions. Metabolomics [10] is the study of the composition and variation of metabolic groups, thereby revealing the overall metabolic response and dynamic changes under different conditions. Thus, the results of integrated omics analysis could be useful for understanding the molecular mechanism of *C. albicans* with the rapid proliferation and enhanced toxicity under the space environment.

In this study, we took an integrative proteomic and metabolomic approach to identify the differentially expressed molecules of *C. albicans* carried by the SJ-10 satellite. The result of proteomic and metabolomic could reflect the phenotypic changes of *C. albicans*. To our knowledge, this is the first multi-omics approach to study *C. albicans* under spaceflight environment.

## Results

### Effect of *C. albicans* under space environment

**Fig. 1:**
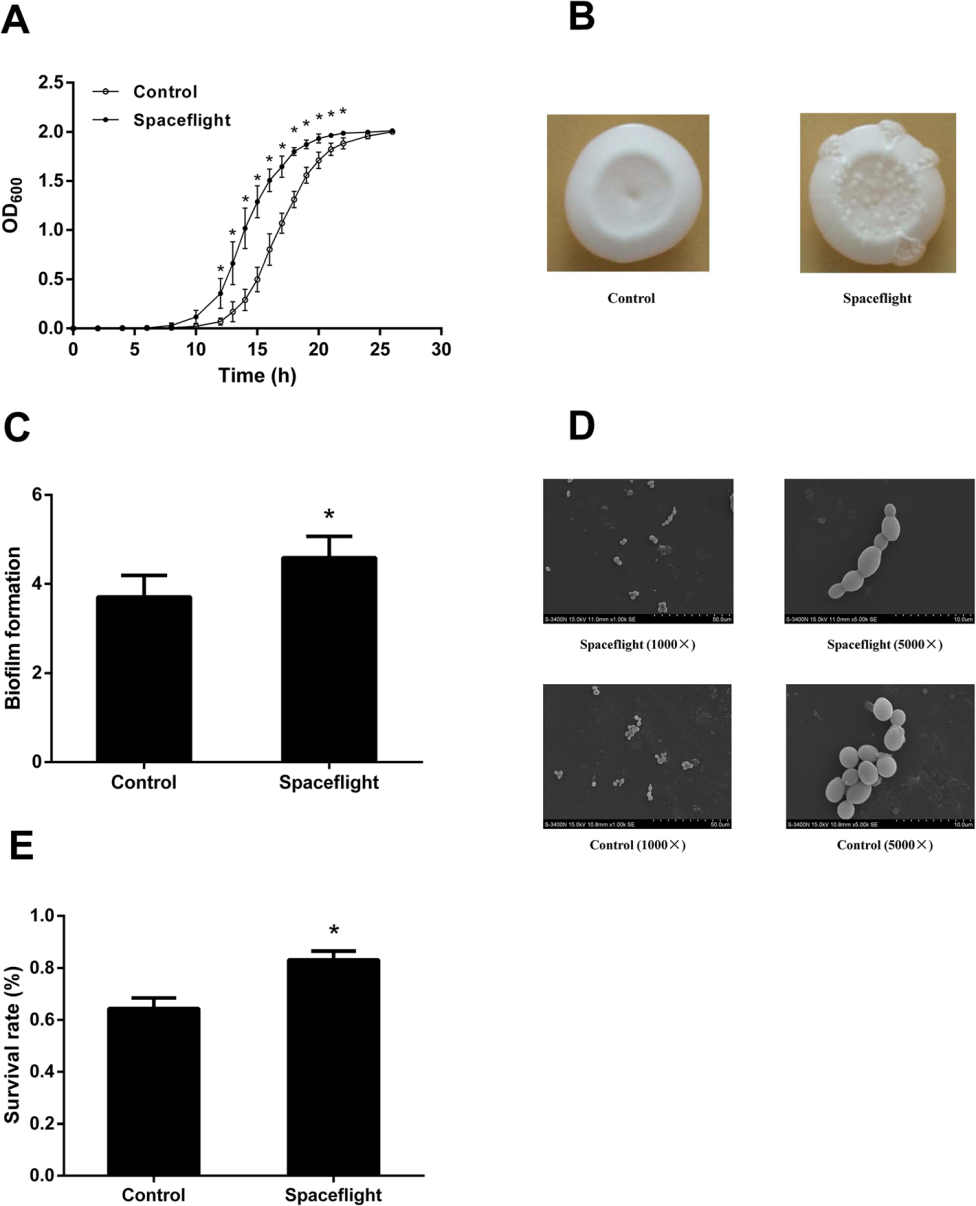
Effect of *C. albicans* under spaceflight environment. **a** The growth curves of *C. albicans* under spaceflight environment compared with control. **b** Colony wrinkles of *C. albicans* after exposured to spaceflight (right) compared with control (left). **c** The relative biofilm formation of *C. albicans*. **d** Cell morphology of *C. albicans* under normal and spaceflight conditions. **e** Survival rate of *C. albicans* exposed to 0.0003% hydrogen peroxide under spaceflight compared with the control group. *: The OD_600_ of spaceflight group was significantly higher than that of control group (*P* < 0.05) at the same time after inoculation

After exposure to the spaceflight environment, *C. albicans* was recovered in the sabouraud-dextrose broth (SDB) medium and their survival was evaluated by OD_600_ measurement. The same measurements were applied towards non-exposed control *C. albicans*, which were cultured at the ground. As shown in Fig. 1A, the growth lag period of the spaceflight group was about 8 h after inoculated into medium, which was shorter than that of the control group (about 10 h). The mean specific growth rate of the spaceflight group was 0.44/h, which was faster than that of the control group (0.35/h, *P* = 0.018). As shown in Fig. 1B, the surface of *C. albicans* in the spaceflight group was rougher and the wrinkles at the edges were increased when compared to the control group. The amount of biofilm formation was significantly increased in the spaceflight group (Fig. 1C). Increased filamentous forms and budding with tight cell connections were observed in spaceflight *C. albicans* through scanning electron microscope (SEM), while grape-like clusters with sparse cell connections were observed in the control group (Fig. 1D). The environmental resistance evaluation result showed that the survival rate of the spaceflight group was higher than that of the control group in the SDB medium containing hydrogen peroxide (Fig. 1E). However, no significant difference was observed between two groups of *C. albicans* in resistance to acid, alkali, alcohol, and salt (Additional file 1: Figure S1). The result of virulence experiment (Additional file 2: Figure S2) showed that the spaceflight group had a lower maximum and a more significant decrease of the normalized cell index (NCI) compared to the control group, which indicates that spaceflight group has stronger cytotoxicity. All in all, the proliferation rate, biofilm formation, antioxidant capacity and cytotoxicity of *C. albicans* were increased after exposure to the spaceflight environment.

### Proteomic analysis of *C. albicans*

**Fig. 2:**
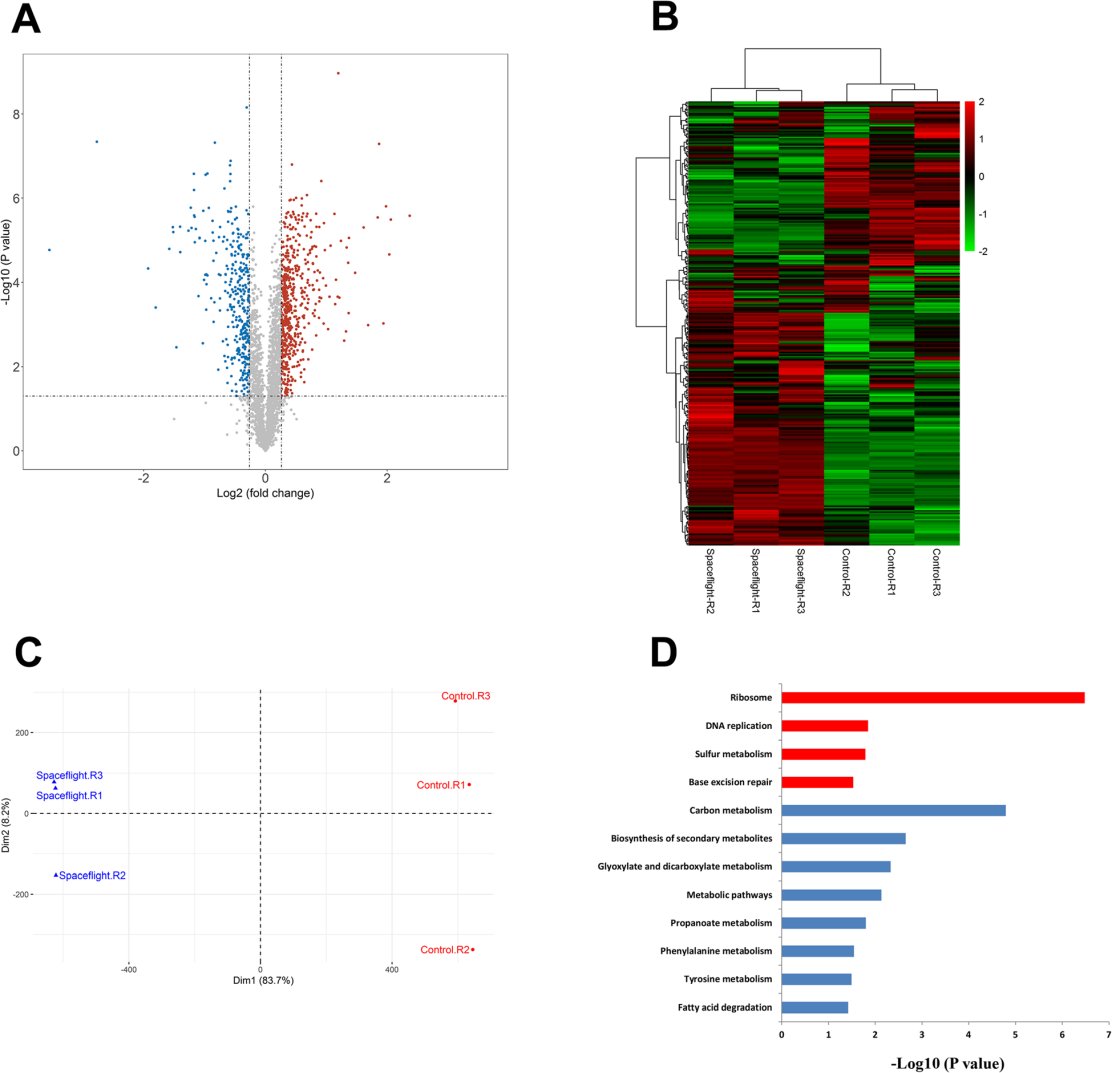
Proteomic analysis of *C. albicans*. **a** Volcano plot of differentially expressed proteins. The red points represented 548 proteins that were up-regulated in spaceflight group (adjusted *P* < 0.05; fold change ≥1.2). The blue points represented 332 proteins that were down-regulated in spaceflight group (adjusted *P* value < 0.05; fold change ≤0.83). **b** Hierarchical clustering of *C. albicans* proteomes. The heat map represented the Z scores of all proteins quantified in TMT technology. **c** PCA analysis of *C. albicans* proteomes. **d** Enriched KEGG pathways in up-regulated (red) and down-regulated (blue) proteins of spaceflight group. The x-axis shows the enrichment significance presented with –log10 (*P* value)

In total, 3670 proteins were identified and 3499 proteins were quantified in the spaceflight group and control group of *C. albicans* by tandem mass tag (TMT) (Additional file 4: Table S1). Proteins with ratios spaceflight /control higher or lower than 1.2-fold change with adjusted *P* value < 0.05 were considered to be significantly changed. This resulted in 548 significantly up-regulated and 332 significantly down-regulated proteins in the spaceflight group compared with the control group (Additional file 5: Table S2), which were shown in Fig. 2A. Quantitative proteome data of *C. albicans* samples were used for hierarchical clustering (Fig. 2B) and principal component analysis (PCA) (Fig. 2C). The result showed that the spaceflight group and control group are clearly separated, which reflected the significant change of protein expression in *C. albicans* after exposure to the spaceflight environment.

To identify biological function changes in *C. albicans* under spaceflight environment, KEGG enrichment analysis was performed with the differentially expressed proteins (DEPs) by DAVID. The significantly enriched KEGG pathways (ranked by *P* value) in up-regulated or down-regulated proteins were shown in Fig. 2D. Among the 548 up-regulated proteins in spaceflight group, pathways including ribosome and DNA replication were enriched, which explains the increased proliferation rate of spaceflight *C. albicans*. In addition, base excision repair was also enriched, which related to DNA damage repair and may explain the increased antioxidant capacity of the spaceflight group. Sulfur metabolism was enriched in up-regulated proteins. While metabolic processes such as carbon metabolism, biosynthesis of secondary metabolites, glyoxylate and dicarboxylate metabolism, propanoate metabolism, phenylalanine metabolism, tyrosine metabolism, and fatty acid degradation were significantly enriched in the down-regulated proteins. This reflected the complex metabolic regulation in spaceflight *C. albicans*.

### Metabolomic analysis of *C. albicans*

**Fig. 3:**
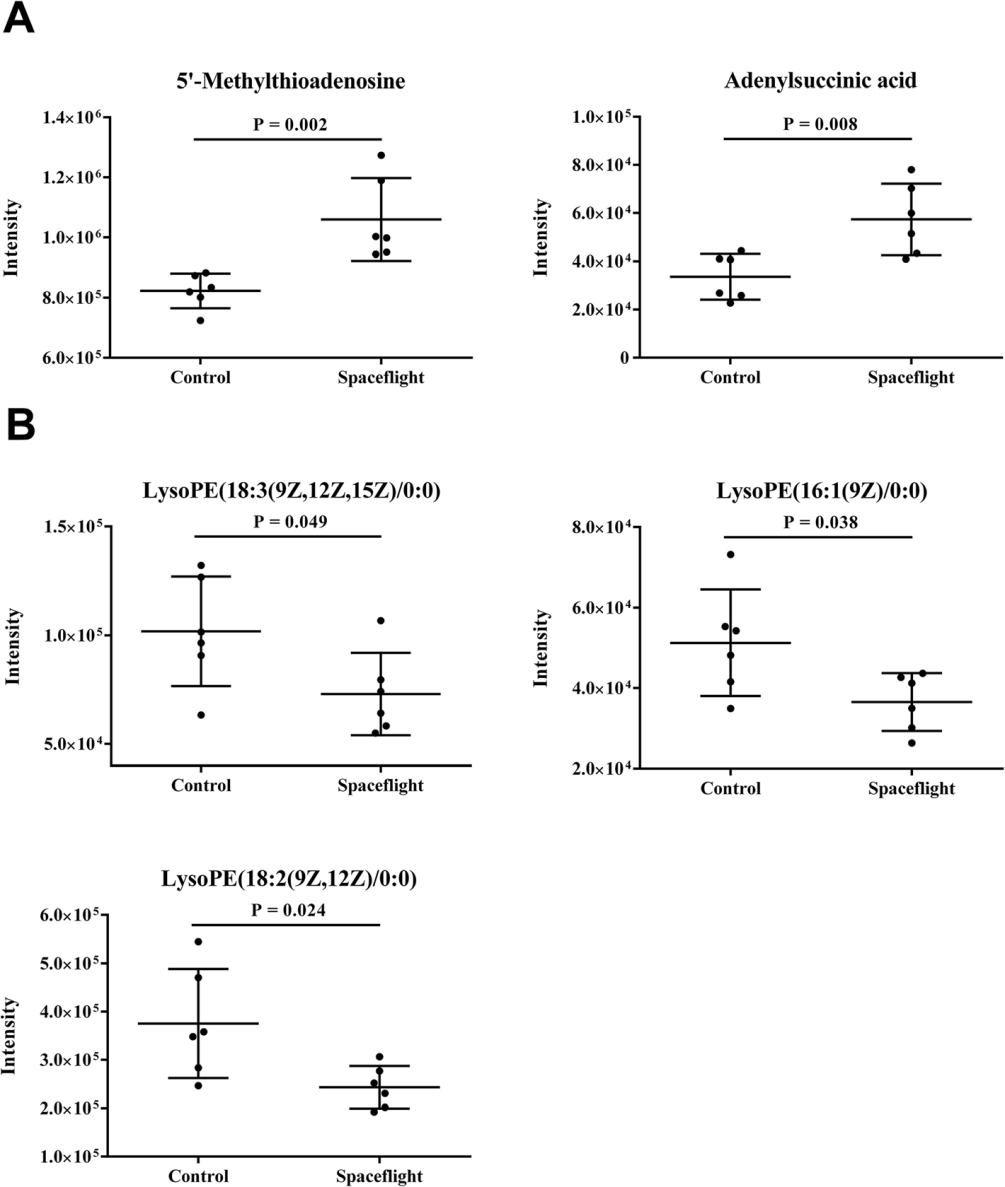
Differential metabolites of *C. albicans*. **a** Scatter plots of two up-regulated metabolites in the spaceflight group. **b** Scatter plots of three down-regulated metabolites in the spaceflight group. *P* value was calculated with unpaired Student′s t-test

To investigate the effect of spaceflight on metabolism, we used ultra-performance liquid chromatography mass spectrometry (UPLC/MS)-based metabolomics approach to untargeted quantify the metabolites in *C. albicans*. In total, 1465 peaks were identified in the spaceflight group and control group of *C. albicans*. After comparing two groups of *C. albicans* with OPLS-DA, five significantly different abundance features (*P* < 0.05, VIP > 1) were identified (Additional file 6: Table S3). Those features were further annotated with public databases. Out of these five metabolites, 5′-Methylthioadenosine and adenylsuccinic acid were significantly up-regulated in the spaceflight group (Fig. 3A). While LysoPE (18:3(9Z,12Z,15Z)/0:0), LysoPE (16:1(9Z)/0:0) and LysoPE (18:2(9Z,12Z)/0:0) were significantly down-regulated in spaceflight group (Fig. 3B).

### Integrated analysis of proteome and metabolome data in *C. albicans*

To integrate the result of proteome and metabolome data, we transformed protein ID and metabolites name to KEGG ID and KEGG compound, respectively. After mapping molecular objects to KEGG pathway, three pathways including purine metabolism, cysteine and methionine metabolism, and alanine, aspartate glutamate metabolism were found to be regulated by both DEPs and differentially expressed metabolites (DEMs) (Additional file 3: Figure S3). Among those three pathways, adenylsuccinic acid, which was up-regulated in spaceflight group, was mapped both in alanine, aspartate glutamate metabolism, and purine metabolism pathway. Consistent with the metabolome result, adenylosuccinate synthetase, which catalyzes IMP and L-aspartate to generate adenylsuccinic acid, was also up-regulated (*P* = 0.0001, fold change = 1.17) in spaceflight group from proteome data. 5′-Methylthioadenosine (MTA) is a naturally occurring sulfur-containing nucleoside, which is a major by-product of polyamine biosynthesis involved in cysteine and methionine metabolism pathway [11]. We found MTA was up-regulated in the spaceflight group with metabolomics analysis. While S-methyl-5′-thioadenosine phosphorylase (MEU1), which involved in the breakdown of MTA and responsible for the first step in the methionine salvage pathway after MTA has been generated from S-adenosylmethionine [12], was significantly up-regulated in spaceflight group of proteomics.

## Discussion

*C. albicans* is an opportunistic pathogenic yeast [13], which usually exists as a commensal organism but can become pathogenic in immunocompromised individuals under a variety of conditions [14]. Long term spaceflight with microgravity and motionless may have adverse effects on the immune system of astronauts [7]. Meanwhile, Jiang et al. [15] reported that simulated microgravity promoted the growth rate of *C. albicans* significantly. Altenburg et al. [16] found that the growth of *C. albicans* in simulated microgravity results in an increase in filamentous forms of the organism, which is consistent with enhanced pathogenicity. Those reports indicated that the existence of *C. albicans* in the space environment might be threated to the health of astronauts. And the specific mechanisms of *C. albicans*’ increased proliferation and pathogenicity need further study. In this study, the combination of proteomic and metabolomic approach was used to study the effects of spaceflight on *C. albicans*. To our knowledge, this is the first report of *C. albicans* under spaceflight conditions with proteomic and metabolomic analyses.

We explored the phenotypic change of *C. albicans* after exposure to the spaceflight environment. Increased proliferation rate and filamentous morphology of *C. albicans* in spaceflight groups were observed, which were consistent with the previous report [15, 16]. In addition, we found that the biofilm relative formation, antioxidant capacity, and cytotoxicity of *C. albicans* were increased in the spaceflight group. Crabbé et al. [17] reported that genes involved in oxidative stress resistance were up-regulated in spaceflight cultured *C. albicans* with microarray analysis, which was consistent with our result. However, Hammond et al. [18] reported that the virulence of *C. albicans* to kill wild type *Caenorhabditis elegans* was reduced under spaceflight environment. The difference between their results and ours may because their experiment was done in the International Space Station with 48 h, while our spaceflight environment was low earth orbit flight for 12 days. The difference in space flight time and environment may have different influences on the microorganism.

Metabolomics only identified five DEMs. There may be two reasons for this result. First, we only used the LC-based approach to detect metabolites, which leads to the missing identification of less polar metabolites. Second, high abundance metabolites such as lipid metabolites were identified, while low-abundance DEMs may be lost. Although our results may lose many metabolites, the five DEMs could be used for further combined analysis. Integrated analysis of proteomic and metabolomic of *C. albicans* revealed that spaceflight may have an influence on cysteine and methionine metabolism pathway. On the one hand, we found that S-adenosylmethionine synthase (SAM2), which catalyzes the formation of S-adenosylmethionine from methionine, was increased in spaceflight group. This was consistent with the up-regulation of MTA, which is synthesized from S-adenosylmethionine. However, the expression of MEU1, which involved in the methionine salvage pathway by breakdown MTA, was increased in the spaceflight group. This may reduce the consumption of methionine. Meanwhile, 5-methyltetrahydropteroyltriglutamate homocysteine methyltransferase (MET6), which involved in the formation of methionine, was also up-regulated (adjust *P* < 0.001, fold change = 1.13). On the other hand, bifunctional cysteine synthase (MET15) [19], which has cysteine synthase activity and may synthase cysteine from serine, was increased in spaceflight group. Meanwhile, aspartate aminotransferase (AAT21) and aspartate transaminase (AAT1), which may play roles in catalyzes cysteine [20], were down-regulated in spaceflight group. So we concluded that cysteine ​​and methionine were accumulated in the spaceflight group of *C. albicans*. Besides, methionine and cysteine are sulfur-containing amino acids, and proteins involved in sulfur metabolism such as sulfite reductase subunit alpha (MET10), sulfite reductase beta subunit, sulfate adenylyltransferase (MET3) and adenylyl-sulfate kinase (MET14) were simultaneously up-regulated in spaceflight group. Interestingly, many research reported [21, 22] that most genes in methionine and cysteine biosynthesis pathway were up-regulated during biofilm formation. In addition, Li et al. [23] showed that the development of *C. albicans* was enhanced by addition of methionine and cysteine. The result inferred from the proteomic and metabolomic analysis was consistent with the observed influence on phenotypic of *C. albicans* after spaceflight.

## Conclusions

We explored the effect of spaceflight on *C. albicans* by combining proteomic and metabolomic analyses in this study. To our knowledge, this is the first proteomic and metabolomic study on *C. albicans* after spaceflight. Increased proliferation rate, biofilm formation, antioxidant capacity, cytotoxicity, and filamentous morphology were observed in the spaceflight group of *C. albicans* compared to the control group. Proteomic analysis identified 3670 proteins and showed that spaceflight samples and control samples could be separated from proteomic data. Enrichment analysis with DEPs indicated that proteins in the ribosome, DNA replication, base excision repair, and sulfur metabolism were significantly up-regulated, while proteins in many metabolic processes were significantly down-regulated. Metabolomic analyses found five DEMs. The combined analysis of proteomic and metabolomic revealed the accumulation of cysteine ​​and methionine in *C. albicans* after spaceflight. This study showed that proteomic and metabolomic are useful tools that could explain the phenotypic changes of *C. albicans*. And the data in this study will facilitate the future mechanism exploration and disease prevention of *C. albicans*.

## Materials and method

### Strains and growth conditions

*C. albicans* (CMCC(F)98,001) were purchased from China General Microbiological Culture Collection Center. SDB was purchased from Oxoid. *C. albicans* was cultured in exponential phase (OD_600_ = 1) and inoculated into SDB. After incubated at 30 °C for 30 h, half of the samples were cultured in space for 12 days carried by the “SJ-10” satellite. And the rest samples were cultured at the ground as control. Strains were preserved by adding glycerin and stored at − 20 °C. Due to the limitations of the cycle and conditions of spaceflight experiments, all replicates were obtained by the recovery of experimental strain.

### Growth curves of *C. albicans*

*C. albicans* in the spaceflight group and control group were recovered on the SDB medium at 30 °C overnight, respectively. Samples were inoculated into SDB liquid medium and incubated at 150 rpm at 30 °C. The growth curves of *C. albicans* were recorded by using a spectrophotometer. The initial OD_600_ measurement is measured every 2 h at the beginning of the lag phase and is measured every 1 h in the exponential phase. Specific growth rate was calculated as the max value of Δlog10(OD_600_)/ Δtime in the exponential phase.

### Colony wrinkles and biofilm formation assay

Preserved strains of *C. albicans* (5 μL) were inoculated into SDB solid medium and incubated at 30 °C for five h. Colony wrinkles of *C. albicans* were observed with a microscope.

Preserved strains of *C. albicans* (20 μL) were added into 2 mL SDL liquid medium in glass tubes and incubated at 150 rpm at 30 °C until the stationary phase. OD_600_ was measurement after incubation and the glass tubes were washed two times with deionized water gently. Glass tubes were added with 5 mL 0.1% crystal violet solution and dyeing for 15 min after drying 1 h at 60 °C. After washed with deionized water gently, 5 mL dimethyl sulfoxide was added into the glass tubes and OD_570_ was measured 1 h later. Biofilm formation was calculated as 100 × OD_570_/OD_600_.

### Morphology of *C. albicans*

Preserved strains of *C. albicans* (1 μL) were recovered with 5 mL SDB liquid medium at 150 rpm at 30 °C overnight. Then coverslips were placed in a solution mixed with sample and SDB liquid medium (1:2). After incubation 8 h at 30 °C, coverslips were fixed with 5% glutaraldehyde solution at 4 °C overnight. Then samples were sent to the China Academy of Chinese Medical Sciences for SEM analysis. Briefly, samples were fixed with tannic acid for 1 h. Then samples were dehydrated by a graded series of ethanol (50, 70, 90, and 100%) for 15 min at each step, and transferred to tert-butanol for 30 min. In the end, the samples were dehydrated with dryer and coated with gold-palladium. The morphology of *C. albicans* was observed in Hitachi S-3400 N SEM.

### Environmental resistance assay

Preserved strains of *C. albicans* (1 μL) were inoculated into SDB solid medium and incubated at 30 °C overnight. Recovered samples were inoculated into SDB liquid medium and incubated at 150 rpm at 30 °C until the exponential phase (OD_600_ = 1). Then samples were added into five SDB liquid mediums (1:100) containing HCL (pH 3.5), ammonia (pH 9.5), 3% ethanol, 0.0003% hydrogen peroxide or 450 mmol/L NaCl, respectively. While normal SDB liquid medium was used as a background. After incubated 7 h at 200 rpm at 30 °C, samples were diluted with PBS at three volume ratios (10^− 3^, 10^− 4^ and 10^− 5^). And 200 μL diluted samples were evenly spread to SDB solid medium, respectively. The number of colonies was calculated after incubation at 30 °C. The survival rate was calculated as the mean number of colonies from mixed SDB divide by the mean number of colonies from normal SDB.

### Virulence of *C. albicans*

LoVo cells were maintained in DMEM-F12 growth medium containing 10% FBS at 37 °C in a CO_2_ incubator. Cells were harvest with trypsin and adjusting concentration to 2 × 10^5^ cells/mL. *C. albicans* was recovered with SDB liquid medium at 150 rpm at 30 °C overnight. After centrifugation at 4000 rpm for 5 min, the precipitation was resuspended with PBS. After centrifugation again, the precipitation was resuspended with DMEM-F12 medium at the concentration of 4.5 × 107 CFU/mL. Then 100 μL *C. albicans* were added into 200 μL LoVo cells. Samples were detected with real time cell analyzer (RTCA) in a CO_2_ incubator for 10 h at 37 °C.

### Sample preparation for proteome and metabolome analysis

*C. albicans* in spaceflight group and control group were recovered on SDB solid medium at 30 °C overnight, respectively. Recovered samples were inoculated into SDB liquid medium and incubated at 150 rpm at 30 °C and grown to an OD_600_ of 1. Samples were harvested by centrifugation at 4000 rpm for 5 min at 4 °C. The precipitate was collected and washed with PBS three times. All samples were stored at − 80 °C until use.

### Proteome analysis

Samples were resuspended with lysate buffer (7 M urea, 2 M thiourea, 40 mM DTT, 1 mM PMSF [24]) and were sonicated (1 s/1 s intervals, 80 W power) for 3 min. Cell debris was removed by centrifugation at 3000 g for 5 min at 4 °C. Protein concentration was determined by Bradford assay and aliquoted to store at − 80 °C. For each group, 100 μg of proteins were mixed with 120 μL reducing buffer (10 mM DTT, 8 M Urea, 100 mM triethylammonium bicarbonate (TEAB), pH 8.0) in Amicon® Ultra-0.5 Centrifugal Filter (10 kDa) [25] and incubated at 60 °C for 1 h. Then iodoacetamide was added to the solution with the final concentration of 50 mM and incubated for 40 min at room temperature in the dark. After centrifugation at 12000 rpm for 20 min, samples were washed three times with TEAB and digested with trypsin (Promega, Madison, WI, USA) (enzyme to protein ratio 1:50) at 37 °C overnight. Digested peptides of three technical replicates per group were labeled with TMT reagents (Thermo Fisher Scientific) according to the manufacturer’s instructions. For 6-plex TMT, spaceflight group samples were labeled with TMT tags 126, 127, 128, and control samples were labeled with TMT tags 129, 130, and 131, respectively. Equal amounts of TMT-labeled peptides were mixed and dried, then resuspended in buffer A (2% acetonitrile, 98% water with ammonia at pH 10) and fractionated to 15 fractions with 1100 HPLC System (Agilent Technologies, USA).

Peptides were redissolved with 0.1% formic acid (FA) and analyzed on a Q-Exactive HF mass spectrometer (Thermo Fisher Scientific, USA) coupled with a nanospray Flex source (Thermo Fisher Scientific, USA). Samples were loaded and separated by a C18 column (15 cm × 75 μm) on an EASY -nLC™ 1200 system (Thermo Fisher Scientific, USA). The flow rate was 300 nL/min and linear gradient was 90 min. The mass spectrometer was operated in the data-dependent mode with positive polarity at electrospray voltage of 2 kV. Full scan MS spectra (m/z 300–1600) were acquired in the orbitrap with the resolution as 70 K, the automatic gain control (AGC) target was 1e6 and the maximum injection time was 80 ms. The top 10 intense ions were isolated for HCD MS/MS fragmentation. In MS2, the resolution was 17,500 and the AGC target was 2e5. Fragmentation was performed with normalized collision energy (NCE) of 32% and dynamic exclusion duration of 15 s.

The mass spectrometry (MS) raw data were analyzed with Proteome Discoverer software (version 2.2) using the Sequest search engine to search against the UniProt *C. albicans* database. The following parameters were applied: precursor mass tolerance was 10 ppm; fragment tolerance was 0.02 Da; the dynamic modifications were oxidation (M); the static modification was carbamidomethyl (C) and TMT labeling of amines and lysine; a maximum of two missed cleavages was allowed. Peptides with FDR < 0.01 (based on the target-decoy database algorithm [26]) were used for protein grouping.

### Metabolome analysis

Metabolites were extracted with 300 μL methanol and ultrasonic oscillation for 10 min. The supernatant was collected after centrifugation at 12000 rpm for 10 min at 4 °C. UPLC-QTOF/MS (Agilent Technologies, USA) was implemented for metabolites detection with six technical replicates. The experimental quality was evaluated by quality control (QC) samples. Features alignment, picking, and identification were performed by Progenesis QI (Waters, Nonlinear Dynamics, Newcastle, UK). By combining the univariate and multivariate statistical analysis, significantly changed features (*P* value< 0.05, VIP > 1) were acquired. Those features were further annotated by Progenesis QI with public databases including Metlin, LIPID MAPS, PubChem, YMDB (Yeast Metabolome Database) and KEGG.

### Statistical analysis

Perseus software [27] was used to calculate the DEPs with unpaired Student′s t-test. Multiple hypothesis testing is performed using Benjamini–Hochberg correction at 5% significance level for both DEPs calculations and subsequent KEGG enrichment analysis. SIMCA (Version 14.1) was used for statistical analysis of metabolomics data with OPLS-DA (orthogonal projections to latent structures discriminant analysis). For other results, SPSS software package (Version 16.0) was used for statistical analysis with unpaired Student′s t-test. The data are presented as means ± SD. *P* < 0.05 was defined as statistical significance.

## Supplementary information

**Additional file 1: Figure S1.** Survival rate of *Candida albicans* under spaceflight environment compared with control. (A) *Candida albicans* cultured in SDB liquid mediums with HCL (pH 3.5), (B) ammonia (pH 9.5), (C) 3% ethanol, or (D) 450 mmol/L NaCl.

**Additional file 2: Figure S2.** Virulence of *Candida albicans* toward LoVo cells in spaceflight and ground control. Recovered *Candida albicans* was added into LoVo cells and the samples were detected with real time cell analyzer. Normalized cell index (NCI) was used to estimate the percentage change in adhesion, which reflected the virulence of *Candida albicans*.

**Additional file 3: Figure S3.** Integrated analysis of proteome and metabolome result on KEGG pathway. (A) Purine metabolism. (B) Alanine, aspartate glutamate metabolism. (C) Cysteine and methionine metabolism. Boxes represented for proteins and dots represented for chemical compounds. The red color represented proteins or metabolites that were up-regulated in spaceflight group. The blue color represented proteins or metabolites that were down-regulated in spaceflight group.

**Additional file 4: Table S1.** List of proteins identified and quantified with TMT.

**Additional file 5: Table S2.** List of differentially expressed proteins between the spaceflight and control group.

**Additional file 6: Table S3.** List of differentially expressed metabolites between the spaceflight and control group.

## Data Availability

The mass spectrometry proteomics data have been deposited to the ProteomeXchange Consortium (http://proteomecentral.proteomexchange.org) via the iProX partner repository [28] with the dataset identifier PXD016983.

## Abbreviations

AAT1: Aspartate transaminase
AAT21: Aspartate aminotransferase
AGC: Automatic gain control
MET10: Sulfite reductase subunit alpha
MET14: Adenylyl-sulfate kinase
MET15: Bifunctional cysteine synthase
MET3: Sulfate adenylyltransferase
MEU1: S-methyl-5′-thioadenosine phosphorylase
MS: Mass spectrometry
MTA: 5′-Methylthioadenosine
NCE: Normalized collision energy
NCI: Normalized cell index
OPLS-DA: Orthogonal projections to latent structures discriminant analysis
PCA: Principal Component Analysis
RTCA: Real time cell analyzer
SDB: Sabouraud dextrose broth
SEM: Scanning electron microscope
TMT: Tandem mass tag
UPLC/MS: Ultra-performance liquid chromatography mass spectrometry
YMDB: Yeast Metabolome Database
