# Supplementary material for: Integrated proteomic and metabolomic analysis to study the effects of spaceflight on Candida albicans
Source: BMC Genomics. 2020 Jan 17;21:57. doi: 10.1186/s12864-020-6476-5 (PMC6969454; doi:10.1186/s12864-020-6476-5)
Supplement: Supplementary file 1 — Additional file 1: Figure S1. Survival rate of Candida albicans under spaceflight environment compared with control. (A) Candida albicans cultured in SDB liquid mediums with HCL (pH 3.5), (B) ammonia (pH 9.5), (C) 3% ethanol, or (D) 450 mmol/L NaCl. [file 12864_2020_6476_MOESM1_ESM.docx]

**
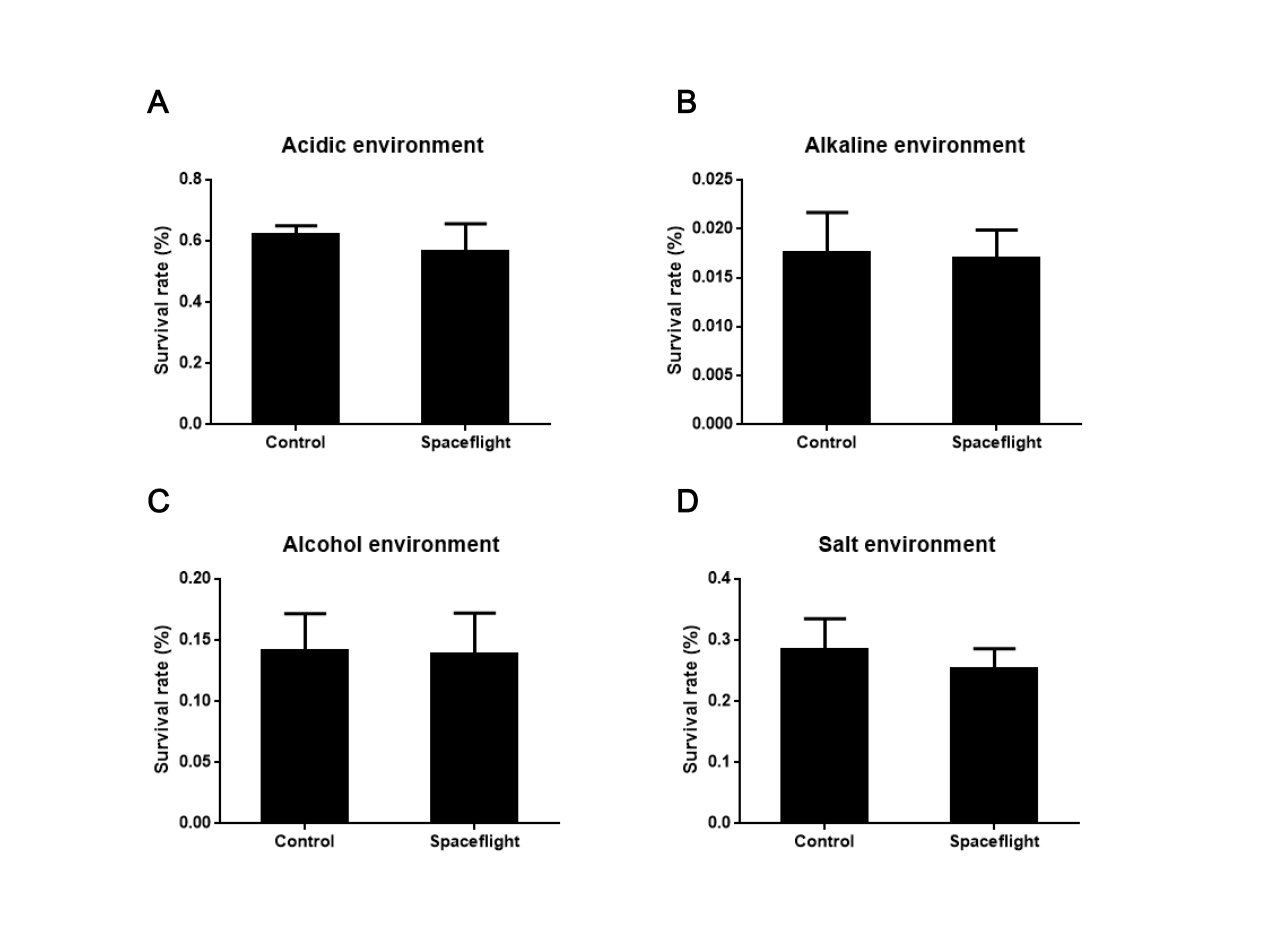
**

**Figure S1. Survival rate of *Candida albicans* under spaceflight environment compared with control.** (A) *Candida albicans* cultured in SDB liquid mediums with HCL (pH 3.5), (B) ammonia (pH 9.5), (C) 3% ethanol, or (D) 450mmol/L NaCl.
