# Supplementary material for: Integrated proteomic and metabolomic analysis to study the effects of spaceflight on Candida albicans
Source: BMC Genomics. 2020 Jan 17;21:57. doi: 10.1186/s12864-020-6476-5 (PMC6969454; doi:10.1186/s12864-020-6476-5)
Supplement: Supplementary file 2 — Additional file 2: Figure S2. Virulence of Candida albicans toward LoVo cells in spaceflight and ground control. Recovered Candida albicans was added into LoVo cells and the samples were detected with real time cell analyzer. Normalized cell index (NCI) was used to estimate the percentage change in adhesion, which reflected the virulence of Candida albicans. [file 12864_2020_6476_MOESM2_ESM.docx]

**
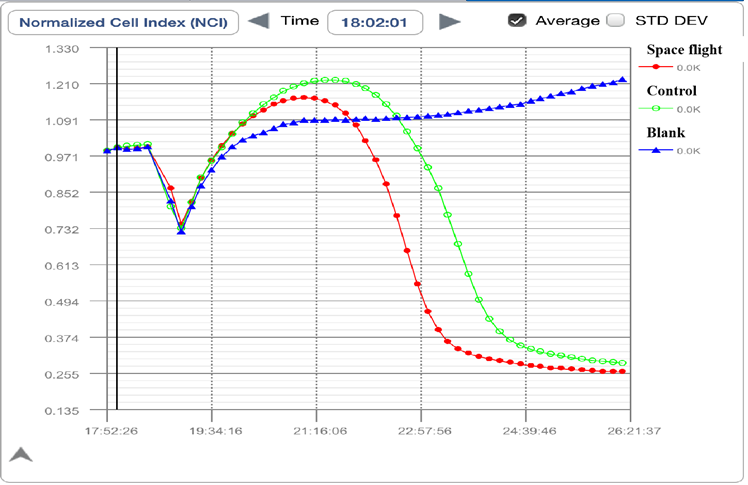
**

**Figure S2. Virulence of Candida albicans toward LoVo cells in spaceflight and ground control.** Recovered Candida albicans was added into LoVo cells and the samples were detected with real time cell analyzer. Normalized cell index (NCI) was used to estimate the percentage change in adhesion, which reflected the virulence of Candida albicans.
