# Supplementary material for: Integrated proteomic and metabolomic analysis to study the effects of spaceflight on Candida albicans
Source: BMC Genomics. 2020 Jan 17;21:57. doi: 10.1186/s12864-020-6476-5 (PMC6969454; doi:10.1186/s12864-020-6476-5)
Supplement: Supplementary file 3 — Additional file 3: Figure S3. Integrated analysis of proteome and metabolome result on KEGG pathway. (A) Purine metabolism. (B) Alanine, aspartate glutamate metabolism. (C) Cysteine and methionine metabolism. Boxes represented for proteins and dots represented for chemical compounds. The red color represented proteins or metabolites that were up-regulated in spaceflight group. The blue color represented proteins or metabolites that were down-regulated in spaceflight group. [file 12864_2020_6476_MOESM3_ESM.docx]

**A**


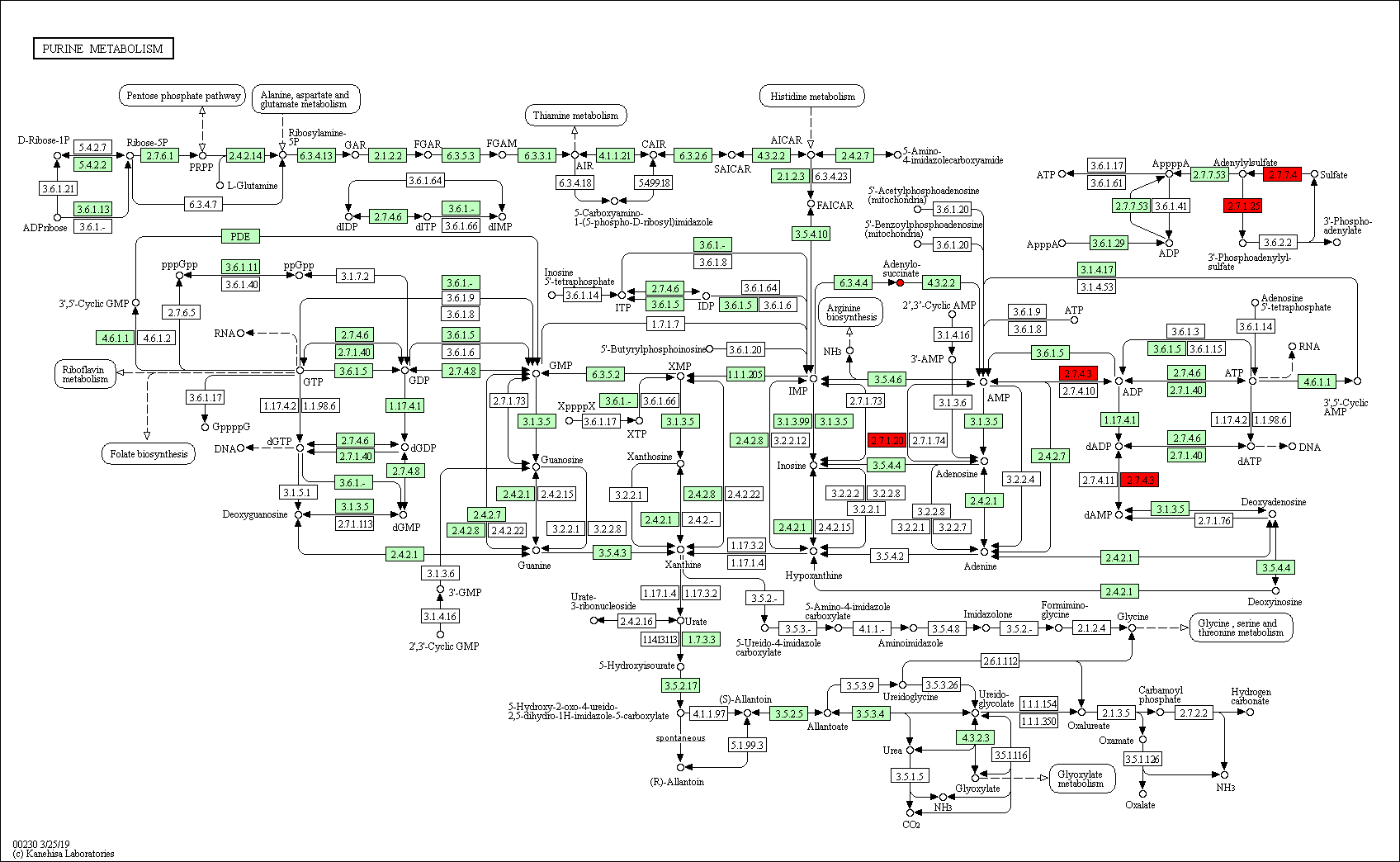


**B**

**
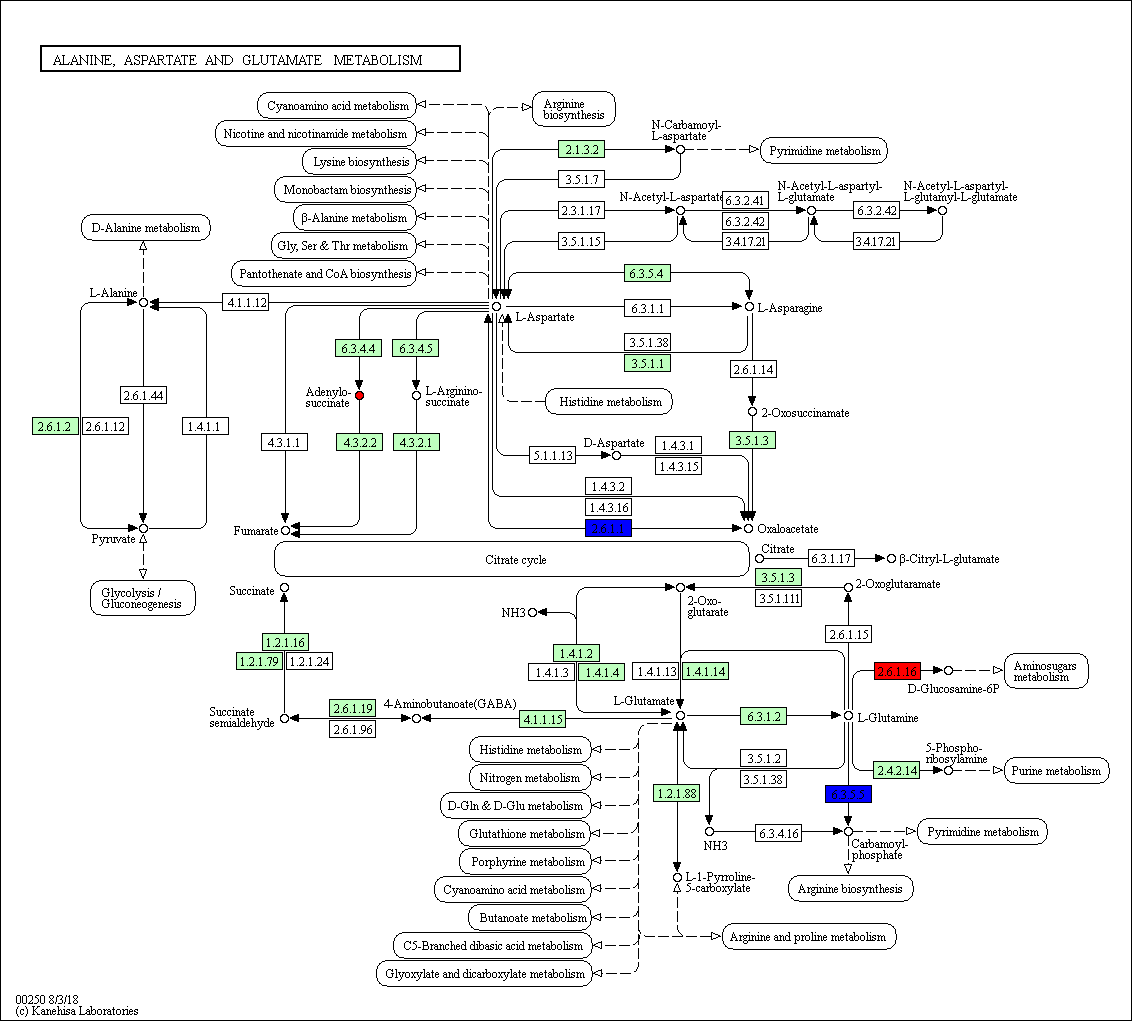
**

**C**

**
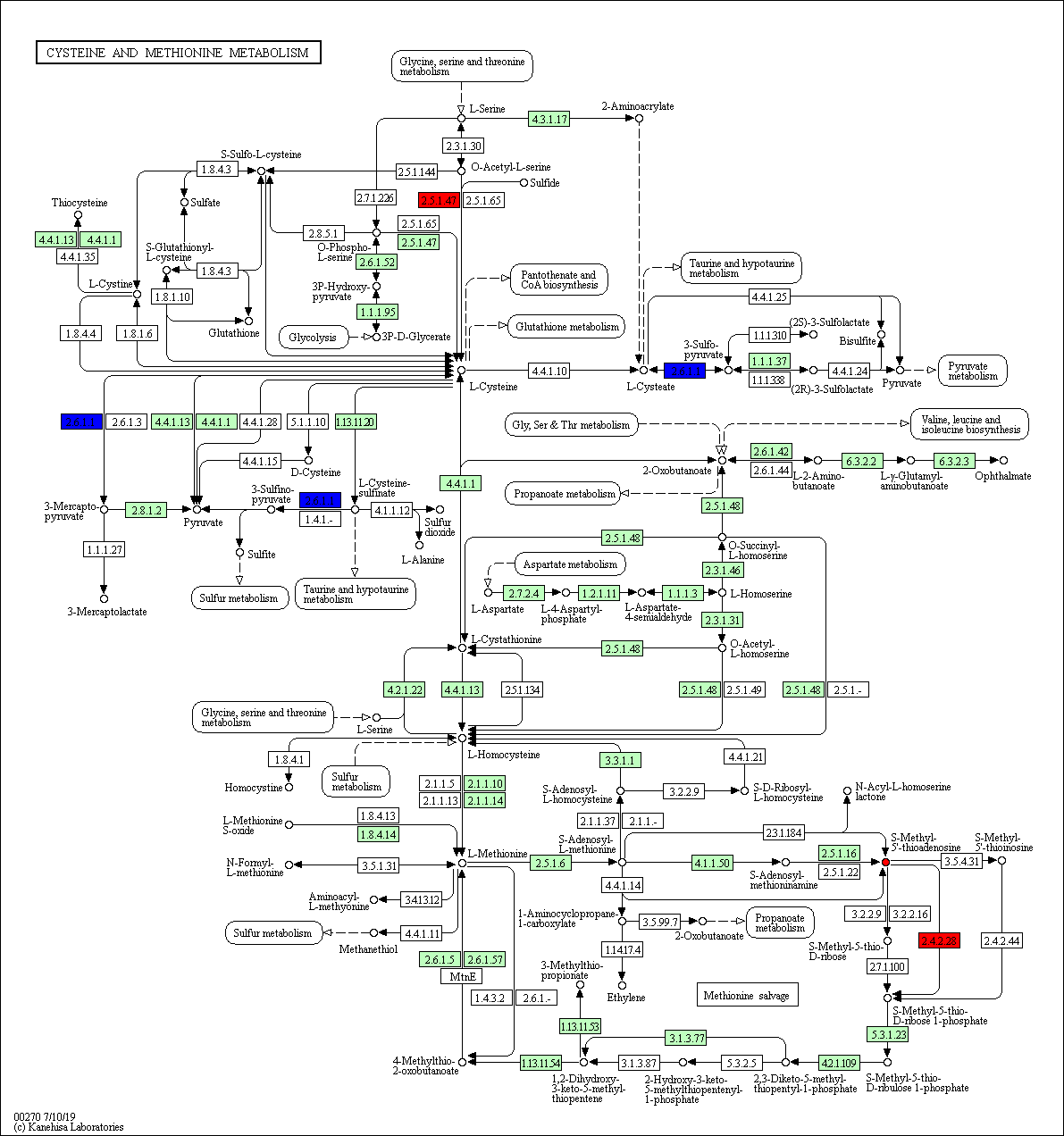
**

**Figure S3. Integrated analysis of proteome and metabolome result on KEGG pathway.** (A) Purine metabolism. (B) Alanine, aspartate glutamate metabolism. (C) Cysteine and methionine metabolism. Boxes represented for proteins and dots represented for chemical compounds. The red color represented proteins or metabolites that were up-regulated in spaceflight group. The blue color represented proteins or metabolites that were down-regulated in spaceflight group.
